# Prevalence and Characteristics of Adolescents with Autism Spectrum Disorder in the New York-New Jersey Metropolitan Area

**DOI:** 10.1007/s10803-023-06058-8

**Published:** 2023-08-29

**Authors:** Walter Zahorodny, Josephine Shenouda, Kate Sidwell, Michael G. Verile, Cindy Cruz Alvarez, Arline Fusco, Audrey Mars, Mildred Waale, Tara Gleeson, Gail Burack, Paul Zumoff

**Affiliations:** 1 Rutgers University – New Jersey Medical School, 185 South Orange Ave, F-511, Newark, NJ 07103 USA; 2 Rutgers University – School of Public Health, Piscataway, NJ USA; 3 Hunterdon Health, Flemington, NJ USA; 4 Atlantic Health System, Goryeb Children’s Hospital, Morristown, NJ USA; 5 Rutgers University - Robert Wood Johnson Medical School, New Brunswick, NJ USA

**Keywords:** Autism, Prevalence, Adolescents, Public health

## Abstract

**Purpose:**

Almost all epidemiologic studies estimating autism spectrum disorder (ASD) prevalence have focused on school-age children. This study provides the first population-based data on the prevalence and expression of ASD among adolescents in a large US metropolitan region.

**Methods:**

Active multiple source ASD surveillance of adolescents aged 16-years was conducted according to the Autism and Developmental Disabilities Monitoring (ADDM) Network method in a four-county New Jersey metropolitan region. Prevalence estimates are provided, characteristics are described and comparison of the distribution and characteristics of ASD is offered for this cohort, at 8 and 16-years.

**Results:**

ASD prevalence was 17.7 per 1000 (95% CI: 16.3–19.2)]. One-in-55 males and one in 172 females were identified with ASD. High-SES was positively associated with ASD and White adolescents had higher ASD prevalence (22.2 per 1000) than Hispanic adolescents (13.1 per 1000). One in four study-confirmed individuals with ASD did not have an ASD diagnosis. A majority of ASD adolescents (58.8%) had a co-occurring neuropsychiatric disorder. White and High-SES individuals had greater likelihood of co-occurring disorder. The demographic distribution and functional profile of ASD was similar in this cohort at 8 and 16-years.

**Conclusion:**

Approximately one-in-55 adolescents in our area had ASD, in 2014, and one-in-4 16-year-olds with ASD was not diagnosed. A majority (3-in-5) of the adolescents with ASD had a co-occurring neuropsychiatric disorder. ASD under-identification and the high frequency of co-disorders in adolescents with ASD pose significant challenges to care and support.

Epidemiologic studies of Autism Spectrum Disorder (ASD) have mainly focused on school-age children, showing a rise in prevalence over time in the United States (US) and worldwide. Not as much is known about the epidemiology of ASD among adolescents. Findings from the National Health Interview Survey (NHIS) and the National Survey of Children’s Health (NSCH) – US parent-report-based surveys posted ASD estimates of 24.3 per 1000 (Xu, Strathearn, Liu, & Bao, 2018) and 26.5 per 1000 (Xu et al., 2019) for adolescents. Similarly, registry data from Sweden and Denmark indicated rates of 24.6 (Idring et al., 2015) and 23.0 per 1000 (Schendel & Thorsteinsson, 2018). In addition, the registry studies showed rising cumulative incidence, suggesting that a significant number of individuals with ASD in those countries were diagnosed after childhood. In contrast, the NHIS and NSCH indicated similar levels of ASD diagnosis, between childhood and adolescence. A population-based study conducted in a South Carolina region, using an active surveillance method, found equivalent ASD estimates (7.6 per 1000) in a cohort, at 8 and 15 years. Estimates derived from administrative data and national surveys have limitations, including misclassification and underestimation. To date, no studies utilizing an active surveillance method have reported on the expression and prevalence of ASD among adolescents in a populous United States (US) metropolitan area.

While some studies have reported on the frequency of co-occurring neuropsychiatric disorders among adolescents with ASD, most have been based on small or non-representative clinical or convenience samples to determine the frequency of co-occurring disorders in adolescents with ASD (Kirsch et al., 2020; Simonoff et al., 2008).

This study provides ASD prevalence estimates by an active multiple-source method for a large diverse population of adolescents (1998 birth cohort, age: 16-years) residing in metro New Jersey, in 2014. Prevalence is reported by demographic factors including sex, race/ethnicity and socio-economic status (SES). Case information includes intellectual level, severity of impairment, ASD diagnosis and non-ASD developmental, neurologic or psychiatric diagnoses. This study reports the frequency of co-occurring disorders, intellectual disability (ID) and ASD features at 16-years and describes differences in identified prevalence and case characteristics in a cohort of individuals at 8 and 16-years, using consistent definitions and methods (Fig. 1).

**Fig. 1:**
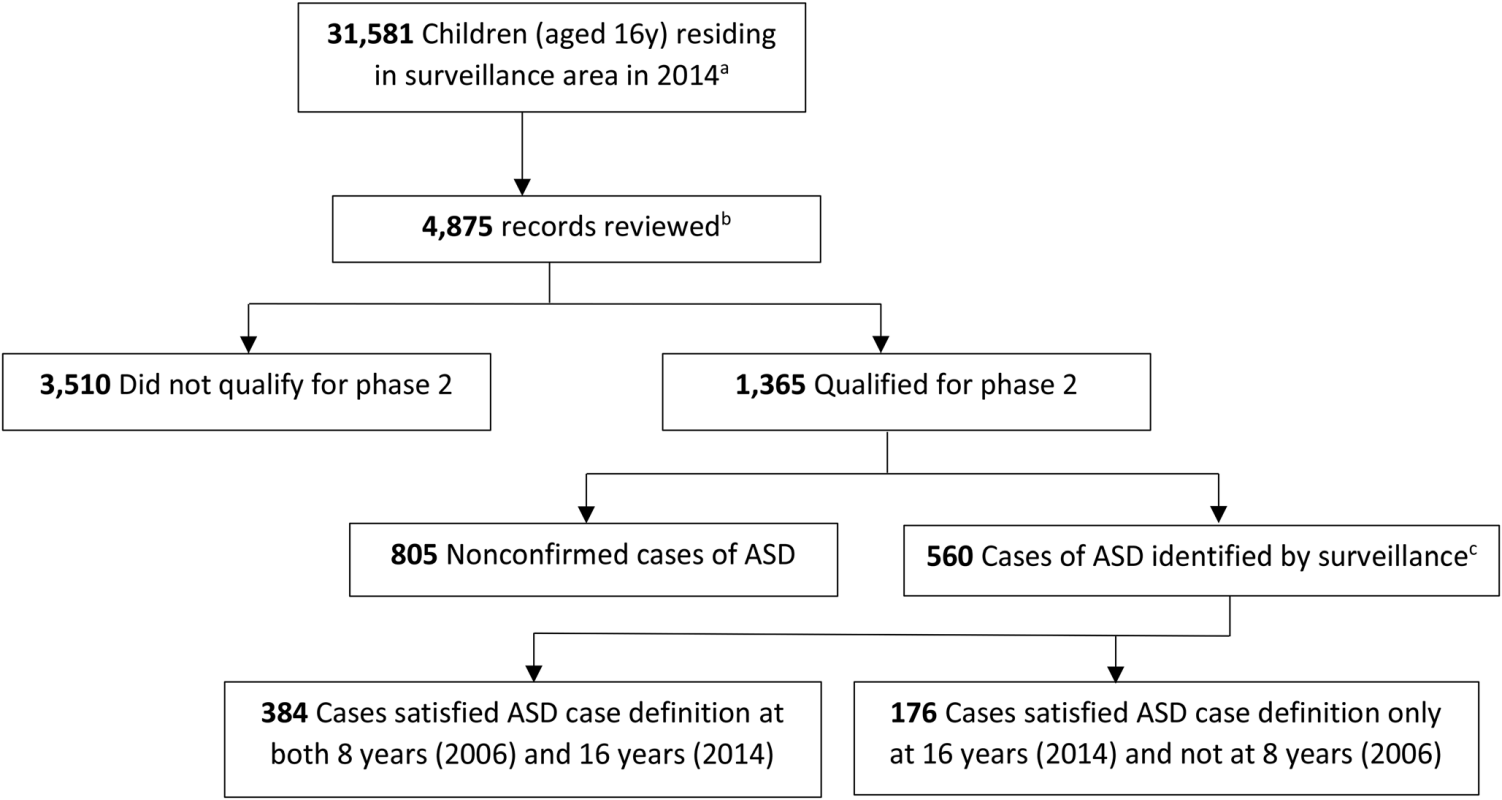
New Jersey Autism Study Surveillance Process, 16-year-olds, Surveillance Cycle 2014. ASD indicates autism spectrum disorder. ^a^Population denominators were obtained from the National Center for Health Statistics. ^b^Approximately 15% of the population qualified for phase 1 of the study based on residency, birth year, receipt of services through special education services in the surveillance year, having 1 or more surveillance-specific International Classification of Diseases, Ninth Revision codes, or some combination of these factors. ^c^Diagnoses of ASD were confirmed by an active surveillance standard case definition based on criteria in the Diagnostic and Statistical Manual of Mental Disorders (Fourth Edition, Text Revision)

## Methods

Active ASD surveillance was conducted according to the Autism and Developmental Disabilities Monitoring (ADDM) Network method for the population born in 1998 and residing in the four county New Jersey surveillance region, in 2014. Case ascertainment was based on comprehensive retrospective review and abstraction of information from multiple health and educational sources (Phase 1), followed by independent scoring and analysis of information by clinician experts, using consistent definitions and reliable procedures (Phase 2) (Rice et al., 2007). The same method was used to determine ASD prevalence and characteristics in this cohort at age 8 (Zahorodny et al., 2014). The study was approved by the Institutional Review Board (IRB) of Rutgers University – New Jersey Medical School. Data were collected between June 2017 and August 2021 and analyzed from December 2021 to September 2022.

In Phase 1, professional evaluations conducted on behalf of educational placement, clinical diagnosis or evaluation, were reviewed using standardized procedures. If an evaluation showed one or more pre-defined ASD signs or triggers (Rice et al., 2007), information was copied into a case-specific, chronologically, organized file. A professional evaluation was defined as an assessment conducted on behalf of educational, developmental, or psychiatric evaluation or services, by a qualified professional. A professional was defined as an individual with specialized education and training in the observation of children with developmental disabilities, including developmental pediatricians, neurologists, psychologists, speech and language pathologists, learning disabilities teaching consultants, occupational and physical therapists and social workers. Researchers reviewed the records of 4875 individuals served in 61 public school districts and 12 hospital-based developmental health centers. 1365 cases showed one or more autism triggers leading to abstraction, analysis and case determination.

In Phase 2, abstracted case information was independently scored and analyzed by clinician experts using a standardized process to determine characteristics and surveillance case status. Confirmed cases included individuals with a documented ASD diagnosis by a community provider and individuals with behaviors and features in professional evaluations consistent with the DSM-IV-TR-based operationalized ASD criteria. Characteristics of ASD cases were described in multiple (median = 9) professional evaluations. Case information was linked to New Jersey birth certificate files to confirm demographic data and to US Census information to obtain median household income (MHI) data, at the census tract level.

### Population & Setting

Individuals born in 1998 and residing in the surveillance region in 2014 were the focus. Ascertainment was conducted in Essex, Hudson, Union and Ocean counties, New Jersey - a densely-populated region within the New York – New Jersey Metropolitan Area. The population was diverse: 42% Non-Hispanic White, 24% Non-Hispanic Black and 29% Hispanic (Table 1) (National Center for Health Statistics (NCHS), 2019). Population denominators were obtained from the National Center for Health Statistics (NCHS) vintage 2019 postcensal bridged-race estimates (National Center for Health Statistics (NCHS), 2019).

**Table 1:** ASD prevalence estimates among children born in 1998 at ages 8-years-old and 16-years-old per 1000

	At age 8-years 2006				At age 16-years 2014				Relative Change
	Pop	ASD Cases	Prevalence per 1000	95% Confidence Interval	Pop	ASD Cases	Prevalence per 1000	95% Confidence Interval	
Overall	30,475	533	17.5	16.1–19.0	31,581	560	17.7	16.3–19.2	1.0 (0.9–1.1)
ASD Diagnosis	30,475	357	11.7	10.6–13.0	31,581	417	13.2	12.0-14.5	1.1 (1.0-1.3)
SPED: Autism Eligibility	30,475	224	7.4	6.5–8.4	31,581	202	6.4	5.6–7.3	0.9 (0.7–1.1)
Sex									
Male	15,471	446	28.8	26.3–31.6	16,033	463	28.9	26.4–31.6	1.0 (0.9–1.1)
Female	15,004	87	5.8	4.7–7.1	15,548	97	6.2	5.1–7.6	1.1 (0.8–1.4)
Race/Ethnicity									
Non-Hispanic White	13,167	278	21.0	18.7–23.6	13,152	292	22.2	19.8–24.9	1.1 (0.9–1.2)
Non-Hispanic Black	7933	116	14.6	12.2–17.5	7671	130	16.9	14.3–20.1	1.2 (0.9–1.5)
Hispanic	7780	112	14.4	12.0-17.3	9038	118	13.1	10.9–15.6	0.9 (0.7–1.2)
SES									
Low	-	-	-	-	15,516	195	12.6	10.9–14.4	-
Mid	-	-	-	-	9234	187	20.3	17.6–23.3	-
High	-	-	-	-	7015	178	25.4	21.9–29.3	-
Intellectual ability									
IQ ≤ 70 (ID)	30,475	194	6.4	5.5–7.3	31,581	198	6.3	5.5–7.2	0.9 (0.7–1.1)
IQ > 70 (non-ID)	30,475	294	9.6	8.6–10.8	31,581	328	10.4	9.3–11.6	1.1 (0.9–1.3)

Prevalence per 1000

Pop = Population denominators were obtained from National Center for Health Statistics postcensal vintage 2019 population estimates for 8-year-olds in 2006 and 16-year-olds in 2014

Relative Change is prevalence ratio comparing 2014 to 2006

SES information was not available for 2006

Abbreviations: ASD = Autism Spectrum Disorder; SES = Socioeconomic Status; SPED = Special Education

### Study Variables

Sex and race/ethnicity data were from source records and confirmed by birth certificate information, as needed. Race and ethnicity were combined and categorized as Non-Hispanic White (White), Non-Hispanic Black (Black) and Hispanic. Socioeconomic status (SES) was represented by median household income (MHI) at the census tract level, using the 2010 and 2014 American Community Survey 5-year estimates (American Community Survey (ACS), 2014). The population counts were estimated by dividing the number of individuals aged 5–9 and 15–19 years, respectively, for each tract, by 5. Tertiles representing Low, Medium and High-SES were based on all New Jersey census tracts. Each ASD case was assigned a level of impairment (Mild, Moderate, Severe), in Phase 2 by clinician experts, based on evaluation of available information and reflecting an overall assessment of the individual’s adaptive, behavioral and social functioning, as well as representing the need for services. Prior to the analytic phase, clinician experts established inter-rater reliability according to the CDC-ADDM standard of > 95% agreement for case definition and > 80% agreement for other features, including impairment level and associated features. Ongoing interrater reliability was tracked and maintained by random, blinded, evaluation of 10% of records. Agreement in case status and feature determination was high across the population (kappa range: 0.8-1.0). The clinician experts satisfied all CDC ADDM training and experience requirements; 4 had > 10-years experience with ADDM methods and 2 experts had > 5-years experience.

Level of intellectual ability was based on most recent IQ test. Intellectual disability (ID) was signified by a Full-Scale (FS) Intelligence Quotient (IQ) score ≤ 70 on the most recent test. Borderline Intellectual Disability (BID) was defined by FS IQ: 71–85, and Average or Above Average IQ was defined by IQ score > 85. In the absence of IQ scores, a qualified examiner’s statement was used to categorize intellectual ability to one of the three specified levels. Information on diagnosed ASD and neuropsychiatric disorders (including ADHD, Anxiety Disorders, Mood Disorders, Conduct Disorders, Seizure Disorder), from birth through 16-years, was recorded from professional evaluations. Special education classification was determined from the most recent individualized educational plan.

### Data Analysis

Population denominators were obtained from the National Center for Health Statistics (NCHS) vintage 2019 postcensal bridged-race estimates (National Center for Health Statistics (NCHS), 2019). Linkage with New Jersey vital records, including birth certificate files, was established to determine individuals who were born in New Jersey and to confirm race/ethnicity information. Prevalence was calculated as the number of individuals, aged 16-years, who satisfied the ASD case definition, divided by the number of individuals, aged 16-years, residing in the surveillance region. Prevalence estimates are reported per 1000 and by sex, race/ethnicity and SES group. Wilson Score method was used to calculate 95% confidence intervals (95% CI). ASD prevalence estimates for the cohort at age 8 were re-calculated using updated US Census data. Pearson Chi square tests were used to test for significance in comparison of proportions. Prevalence ratio and 95% CI were used to compare differences in prevalence estimates at 16 and 8 years. Significance was set at p < 0.05. Statistical analyses were performed using SAS 9.4.

## Results

Five hundred and sixty individuals with ASD were identified, in a population of 31,581 16-year-olds, yielding a point prevalence of 17.7 per 1000 (95% CI: 16.3–19.2). Male-to-female ratio was 4.7 to 1. ASD prevalence was highest among White adolescents (22.2 per 1000; 95% CI: 19.8–24.9) and lowest among Hispanic adolescents (13.1 per 1000; 95% CI: 10.9–15.6). Adolescents with ASD from High-SES tracts (25.4 per 1000; 95% CI: 21.9–29.3) were twice as prevalent, compared to peers from Low-SES tracts (12.6 per 1000; 95% CI: 10.9–14.4) (Table 1). 35% of 16-yearold with ASD had intellectual disability (ID IQ ≤ 70), 17.3% had BID and 41.1% had Average or Above-Average IQ. While there were no differences by sex among adolescents with ASD when considering intellectual ability, there were significant differences by SES and race. Among ASD adolescents residing in high SES areas, 61% had average to above average IQ, compared to 46% residing in Mid SES areas and 35% residing in Low SES areas (p < 0.001). Similarly, 61% of White adolescents with ASD had average to above average IQ, compared to 26% of Black and 39% of Hispanic adolescents.

### Characteristics at Age 16

Among surveillance confirmed cases, 417 (74%) individuals had an ASD diagnosis by 16-years. 59% of 16-year-olds with ASD had one or more documented psychiatric, neurologic or developmental disorders. Most co-diagnosed were: Attention Deficit Hyperactivity Disorder (ADHD), Anxiety and Mood Disorders (Table 2). Seizure Disorder was identified in approximately 7–8%. The likelihood of co-disorders did not vary by sex. White and High-SES adolescents had higher frequency of co-occurring neuropsychiatric disorders, compared to minority peers.

**Table 2:** Differences between ASD cases identified at age 8- and 16-years and ASD cases identified at age 16-years only (n = 560)

	Total		Cases confirmed in 2006 and 2014		Cases confirmed only in 2014	
	n	%	n	%	n	%
Overall	560		384	69%	176	31
*Demographic Factors*						
Sex						
Male	463	83	325	85	138	78
Female	97	17	59	15	38	22
Race/Ethnicity						
Non-Hispanic White	292	52	200	52	92	52
Non-Hispanic Black	130	23	94	24	36	21
Hispanic	118	21	79	21	39	22
Other	20	4	11	3	9	5
SES						
Low	195	35	138	25	57	32
Mid	187	33	118	21	69	39
High	178	32	128	23	50	29
*Clinical Factors*						
Intellectual Ability						
IQ ≤ 70 (ID)	198	35	150	39	48	27
IQ > 70 (non-ID)	328	59	210	55	118	67
Unknown IQ	34	6	24	6	10	6
Degree of impairment						
Mild	246	44	145	38	101	57
Moderate	214	38	155	40	59	34
Severe	100	22	84	22	16	9
ASD Diagnosis	417	74	291	76	126	72
Co-occurring Disorders	329	59	197	51	132	75
ASD + ADHD	211	38	115	30	96	55
ASD + Seizure Disorder	41	7	27	5	14	8
ASD + Mood Disorder	64	11	26	7	38	22
ASD + Anxiety Disorder	97	17	51	13	46	26
Service Factors						
SPED: Autism Eligibility	202	36	160	42	42	24
Special Education	454	81	316	82	138	78
County						
Essex	168	30	129	33	39	22
Hudson	93	17	69	18	24	14
Ocean	139	25	83	22	56	32
Union	160	28	103	27	57	32
Born in New Jersey	426	76	305	79	121	69

Abbreviations: ASD = Autism Spectrum Disorder; ADHD = Attention Deficit Hyperactivity Disorder; SES = Socioeconomic Status;

SPED = Special Education

### Characteristics at 8 and 16-Years

Recalculated prevalence estimation for the cohort, at 8 years, yielded a prevalence rate of 17.5 per 1000 (95% CI: 16.1–19). Prevalence ratios (PR) describing frequency of occurrence by demographic factors at 8 and 16-years, showed no change according to sex or race.

The number of individuals diagnosed with ASD increased from 11.7 to 1000 at age 8 to 13.2 per 1000 at 16, while the number of individuals receiving any special education services between 8 and 16-years decreased, as did the number receiving services under the Autism classification (Table 1).

Three-hundred-and-eighty-four individuals (68.6%) satisfied the ASD surveillance case definition at 8 and 16-years. The impairment profile varied slightly between the two age points. At 8-years, 28% of the cohort had severe impairment, while at 16-years, 22% were severely impaired. Five individuals with ASD had higher (improved) IQ levels at 16, than at 8-years. Fewer individuals received special education services at 16-years and fewer were classified under the Autism classification, than at 8-years. Individuals who met the ASD criteria at age 16, but not at 8 (n = 176), were more likely to have mild or moderate impairment, more likely to have BID or Average IQ (p < 0.02) than IQ ≤ 70 and were more likely to have co-occurring neuropsychiatric disorders (p < 0.001). The proportion of individuals with ASD and Seizure Disorder was similar at 8 and 16-years. At age 16, the cohort included more individuals who migrated to the surveillance region (Table 2).

## Discussion

The findings show that nearly 2% (17.7 per 1000) of 16-year-olds in our region had ASD, similar to the prevalence estimate for the cohort identified at 8-years (17.5 per 1000) (Zahorodny et al., 2014), but lower than estimates from parent-report surveys (Xu et al., 2018) and registries (Idring et al., 2015; Schendel & Thorsteinsson, 2018). We expected an increase in ASD prevalence at older ages, but our findings show a stability in ASD prevalence across ages. Consistent with multiple previous studies, ASD prevalence varied by SES (Durkin et al., 2010; Durkin & Yeargin-Allsopp, 2018; Thomas et al., 2012) and Hispanics had lower levels of identified ASD, compared to White adolescents, possibly reflecting a disparity in ASD detection. Black and Hispanic adolescents with ASD were more likely to have co-occurring ID, compared to White adolescents.

A high burden of co-occurring neuropsychiatric disorders was evident. Nearly 3-in-5 16-year-olds with ASD had one or more co-occurring disorder. Approximately 40% had ADHD, 35% were intellectually disabled and 28% had a Mood or Anxiety Disorder. Studies have shown that individuals with ASD and ADHD have higher rates of attentional and emotional deficit and more frequent mood and conduct disorders (Beighley et al., 2013). ASD individuals identified after age 8, were more likely to have co-occurring disorders. At 16-years, one-in-four adolescents identified by surveillance did not have an ASD diagnosis by a community provider.

This snapshot provides a baseline estimate of ASD among adolescents in a diverse US metropolitan area. Ongoing, population-based monitoring is necessary to understand the changing prevalence of ASD, as well as to guide educational and health services planning. Autism frequently co-occurs with other disorders, which complicates evaluation, treatment and outcomes. The number of adolescents and young adults with ASD is significant and this growing minority will require a range support and care, over time.

### Limitations

Individuals not receiving special education and persons not attending public school, as well as those receiving clinical care from private or out-of-region providers, may not have come under review, possibly leading to underestimation. This study was conducted in a US metropolitan region and the findings may not be generalizable to other areas. The migration history of cases could not be reliably determined, reducing the ability to interpret new and missing cases between the time points. Numerous contextual and process-oriented factors, including timing of identification are not provided by this study.

## Conclusion

In this 1998-born cohort residing in a US metro area, ASD affected one-in-56 adolescents and usually co-occurred with additional neuropsychiatric disorders, posing a significant challenge to care. One-in-four individuals with ASD were undiagnosed, even at age 16. Moreover, our evidence suggests persisting race and SES-based differences in autism identification and distribution. The findings demonstrate the overall stability of cohort-specific ASD estimates between childhood and adolescence using the active surveillance method and suggest additional areas and questions for further research.
